# A case report on the placement of a temporal dialysis catheter in the femoral artery for emergency dialysis

**DOI:** 10.22088/cjim.14.4.755

**Published:** 2023

**Authors:** Konstantinos Mavromatidis, Athanasios Athanasios Bakaloudis, Ioannis Skandalos, Irini Kalogiannidou, Anastasia Georgoulidou

**Affiliations:** 1 Department of Nephrology, Renal Unit “Dimokrition”, Komotini, Greece; 2 Renal Unit Gen. Hosp. , Komotini, Greece; 3 Department of Surgery, Gen . Hosp. “St. Pavlos”, Thessaloniki, Greece

**Keywords:** Emergency dialysis, Femoral artery catheterization, Pulmonary edema, External jugular vein

## Abstract

**Background:**

Ensuring vascular access is essential for dialysis patients. This can be achieved through an arteriovenous anastomosis (fistulae), a central venous catheter, or an arteriovenous graft. However, in some cases vascular access to the patient's blood is not possible.

**Case Presentation:**

A multi-vascular male patient, who had been undergoing dialysis for 17 years, was presented to our renal department. There was no possibility of vascular access to the patient’s venous network for dialysis. A peritoneal dialysis catheter was inserted, but it was malfunctioning. An attempt was made to place a HeRO AV Graft, but it did not succeed due to contraindications from the patient's venous network, as shown by the computed tomography.

While trying to solve the problem in order to dialyze the patient during his hospitalization, he experienced severe shortness of breath with tachypnea (pulmonary edema), along with acidosis and hyperkalemia. A temporal dialysis catheter was urgently inserted into the left femoral artery and isolated ultrafiltration was performed, and by removing 1500 ml of ultrafiltration, the patient improved significantly. During the subsequent days, he underwent another 11 dialysis sessions using the femoral artery catheter. While he was hospitalized and being dialyzed via the femoral artery, a successful effort was made to catheterize the right external jugular vein, from which he continues to be dialyzed today.

**Conclusion:**

The patient’s treatment through the placement of a temporal dialysis catheter in the femoral artery enabled him to survive. It is our belief that such a solution could be helpful in similar cases.

Vascular access is a very fine lifeline for dialysis patients (1). It allows access to the vascular network for dialysis, which is necessary for survival. This access can be achieved through an internal arteriovenous anastomosis (fistulae), an arteriovenous graft, or even a double lumen central venous catheter. In fact, for the care of the patient's veins, according to NKF-K/DOQI instructions, the order of choice is the creation of a radial-cephalic arteriovenous anastomosis, a brachial-cephalic to the elbow, and a brachial-basilic anastomosis with superficiality and displacement of the basilic vein (2). In other words, the principle of "first the internal arteriovenous anastomosis and lastly the central venous catheter" applies (3).Today, many patients do not have a satisfactory vascular network, making it difficult to create an internal arteriovenous anastomosis, which is the best solution (4).

This difficulty or even the inability to create such access usually occurs in the elderly, diabetics, and patients who have undergone many hospitalizations for a variety of reasons (resulting in venous lesions from prolonged venipuncture).

In such cases, it is necessary to use a central vein (jugular, femoral, or subclavian) in order to place a dialysis catheter. Currently a catheter is used for vascular access in 1% of dialysis patients in Japan, 18% in the United States, up to 42% in Belgium and 44% in Canada (5).

The use of catheters very often leads to stenosis, but also thrombosis of the veins, resulting in the destruction of a large part of the venous network (6, 7). Thus, from this perspective, in many patients it is difficult to gain access to the vascular network, which makes it impossible to perform dialysis. In these cases, when there is an urgent need for dialysis, accessing an artery with a dialysis catheter may be a temporary solution, providing precious time for a more permanent solution that allows the patient to survive, as was observed in the present case study.

## Case Presentation

A 56-year-old anuric male patient, who had been on a dialysis program since 2003 for four and a half hours per session, three times a week, was hospitalized due to a loss of vascular access. He was a multi-vascular patient with a history of malignant hypertension, myocardial infarction (at 36 years old), and stroke (at 37 years old). He had a damaged venous network from previous arteriovenous anastomosis, and in the past, grafts had been placed in his large veins without the possibility of creating a new means of vascular access (internal arteriovenous, graft, or catheter). Grafts were placed in both his upper arms, and catheters were placed in the right subclavian, jugular (right and left) as well as the femoral veins (right and left), and there were problems with venous thrombosis (a deep and superficial network of the left and right legs), both presenting edema, consecutively. The patient also presented superior vena cava syndrome.

The medications that the patient was taking daily before hospitalization included insulin (insulin lispro-Humalog 200 IUx2 and insulin glargine-Toujeo 40 IUx1) and bemiparin 3500 IU subcutaneously (due to a recent right femoral vein thrombosis). In addition, he was taking clobazam 10 mg (occasionally), along with omeprazole 20 mg, carvedilol 12.5 mg, atorvastatin 20 mg, fenofibrate 145 mg, calcium carbonate 1500 mg, sevelamer 7.2 gr, acetylsalicylic acid 100 mg, paricalcitol 1 mcg, and clopidogrel 75 mg daily, as well as folate calcium (30 mg/week). On July 26, 2020, a peritoneal dialysis catheter was inserted, as peritoneal dialysis was considered the last and perhaps only solution in terms of dialyzing the patient. The plan was to start the method immediately (automated peritoneal dialysis, with the patient in a supine position); however, this was not achieved due to a malfunction of the catheter and the outflow of dialysate from the exit site. However, on July 29, 2020, the patient presented severe dyspnea (due to hypervolemia) and coexisting hyperkalemia (potassium = 6.47 mmol/L), severe uraemia (serum creatinine = 1500 μmol/L) and acidosis (pH = 7.287, PaCO_2_ = 38 mmHg, bicarbonate 18 mmol/L, anion gap = 18.8 mmol/L). At 9:00 pm on the same day, it was decided that a catheter should be placed in the femoral artery and immediately used for isolated ultrafiltration (1500 ml of ultrafiltrate was removed rapidly). The next day, and for three consecutive days, the patient underwent four-hour hemodialysis sessions, and afterwards he began a three-week program of dialysis as he had done in the past (four and a half hours/session). On August 6, 2020, the patient’s uremia (serum creatinine = 884 μmol/L) had clearly improved, as well as his hypervolemia, hyperkalemia, and acidosis. Over a period of 20 days with the femoral artery catheter, 11 dialysis sessions were performed (with the final one on August 14, 2020). It was noted that from the day of the catheter placement until August 6, 2020, the patient did not taken heparin, other than the dose necessary for the dialysis session, nor any other oral anticoagulant (aspirin, clopidogrel) so as to avoid any possible bleeding. (The patient was very bad-tempered and almost never followed medical instructions. After having the catheter placed in the femoral artery, he wandered the corridors and was often spotted in the hospital courtyard, smoking).

Dialysis conditions during that period were as follows: Hydrophilic PEPA filter (polyester-polymer alloy), blood flow ~300 ml/min, filter heparinization with 3500 IU bemiparin/1000 ml NaCl 0.9%, 2000 IU classic heparin intravenously at the beginning of the session followed by 1000 IU of classic heparin/hour, while the catheter lumens were locked with 15% NaCl solution. During the sessions, venous pressure ranged from 120–130 mmHg. Due to the presence of the catheter in an artery (which was not desirable but necessary), as well as the peritoneal catheter malfunctioning (it had a very slow outflow of dialysate and an outflow from its exit site), on August 3, 2020, the patient was referred to a vascular surgeon for placement of a HeRO AV Graft (implant graft with catheter). In particular, a catheter was to be placed in the internal jugular vein, near the right atrium, and then anastomosed with a graft in the brachial or subclavian artery. However, the angiographic examination that took place on the same day revealed that the procedure could not be performed, so the patient returned to our clinic without the HeRO AV Graft. Due to the malfunctioning of the peritoneal dialysis catheter and the inability to place the HeRO AV Graft, on August 13, 2020, the peritoneal catheter was repositioned. Afterwards, it was noted to work successfully, and on August 16, 2020, the patient was sent to a vascular surgeon, who succeeded in placing a permanent dialysis catheter in the right external jugular vein, from which he continued dialysis. On August 21, 2020, we again sent the patient to the vascular surgeon in order to remove the catheter from the femoral artery.

On September 11, 2020, due to pain, coldness in the left leg, and cyanosis of the fingers (ischemia), the patient was admitted to a vascular surgery clinic. Thrombosis was found from the starting point of the common iliac to the starting point of the common femoral artery (non-pulsative femoral artery). Hybrid reperfusion of the left leg was performed under local anesthesia, which included the removal of multiple thrombi from the iliac axis and the stenting of the left common iliac up to the middle third of the left external iliac artery. Five days later, on September 15, 2020, he was discharged from the vascular surgery clinic in excellent condition, considering his general status, with anticoagulant medications (acetylsalicylic acid 100 mg/24 hours and clopidogrel 75 mg/24 hours for two months, followed only by clopidogrel 75 mg/24 hours for the rest of his life).

**Scientific Council: **The study was conducted in accordance with the Helsinki Declaration of Human Rights and the patient signed a consent form.

## Discussion

Permanent venous dialysis catheters are used only in patients with contraindications (e.g., the presence of a pacemaker on the same side) or an inability to create an internal arteriovenous anastomosis (8). There are many potential ways to dialyze patients with end-stage renal disease (ESRD) and central venous occlusion or stenosis; however, when all the veins are depleted, vascular access is particularly difficult. Of course, there are very few examples of the use of an artery as an access point for dialysis; exceptions include those defined by Burger et al. (who published cases of patients presenting damaged main large veins in their arms). In 20 of these rare cases, an axilla-axillary graft was placed, resulting in good graft patency in >90% of the cases over a period of six months (9), a result that was also confirmed by others (10).

We must keep in mind that dialyzed patients with thrombosed or narrowed central veins do not have the ability to wait for vascular surgeons to create new vascular access, which obviously take some time to mature. Furthermore, they may not be able to receive general anesthesia, due to, for instance, a high serum K^+^ levels or the existence of hypervolemia, as was the case with our patient (usually anesthesiologists refuse to give these patients general anesthesia). The catheterization of the femoral artery for emergency dialysis possibly saved the patient's life in an emergency situation (pulmonary edema), and it created precious time to find alternate solutions for the future. Without using the femoral artery, the patient certainly would have died the same day. Obviously, despite the problems that could have resulted from catheterization of the femoral artery, it was necessary and salutary. There was no time to refer our patient to an invasive radiologist or vascular surgeon (as these were not available in our hospital) to try to solve the problem of vascular access. The decision to cannulate the femoral artery was made at nighttime (9:00 pm), when the patient was experiencing shortness of breath and orthopnea, and the doctors were unable to help him with any kind of dialysis (peritoneal or hemodialysis). His consent was sought, as well as that of his wife, and the catheter was placed immediately. Thus, catheterization of the femoral artery was the only (one-way) solution at the time.

It has been noted in the literature that the use of an artery for access for dialysis is safe (11). Dialysis via the femoral artery was performed for the first time in 1969 (12), in two patients in which access for dialysis via any vein was impossible. In those cases, the puncture of the artery (which had become superficial) was performed two to three weeks after the operation, a time period which was necessary for the wound to heal (this is not necessary when a catheter is placed). A heparin-protamine combination was then used for anticoagulation. The mean blood supply during the dialysis session was 280 ml/min, with no problems or complications. After removing the needles, applying pressure for 10–15 min was enough to stop any bleeding from the artery.Frampton et al., from the transplant department at St. George's Hospital in London, published 10 cases over a period of five years, in which temporal dialysis catheters were placed in femoral arteries, because urgent dialysis was needed and there was no other possibility of vascular access (13). Abutaleb also made use of the carotid artery, in addition to the femoral artery, in Saudi Arabia (14). In these studies, dialysis via arteries appeared to be effective and safe.

Of course, the use of arteries for dialysis should be limited, and procedures should be performed only if all other arteriovenous anastomosis or venous positions for the placement of dialysis catheters are non-existent. Thus, Frampton et al. reported in their clinic that catheterization of the femoral artery should be avoided at all costs, and that its use should be allowed only as a last resort in order to perform emergency dialysis, such as in patients with shortness of breath (pulmonary edema) or hyperkalemia (13). Abutaleb also used arteries for emergency dialysis in cases where no other method was feasible (e.g., peritoneal dialysis), including vascular access (venous), and of course when the patients were not candidates for emergency transplantation. The femoral artery was punctured in four of the cases and the carotid artery in one case (for one dialysis session only, after which a peritoneal dialysis catheter was inserted and the patient lived with it for another five years) (14). From these experiences, it seems that puncturing arteries for dialysis provides enough time (in seemingly dead-end cases) to find more acceptable solutions, as was the case with our patient.

It is important to mention that in our patient, there was no possibility of dialysis via a venous dialysis catheter, nor for peritoneal dialysis. In a patient with tachypnea (36 breaths/min), pulmonary edema, hemoglobin saturation with oxygen 91.6% at room air and PaO_2_ = 68.7 mmHg (PaO_2_/FiO_2_ index was 326.97 mmHg, i.e., poor O_2_ diffusion), it was obvious that an effort had to be made for his survival. Hyperkalemia, acidosis, and severe uremia coexisted, but these did not cause the emergency dialysis. Placement of a dialysis catheter in the femoral artery is not a conventional route for dialysis, but it is safe and life-saving for those patients who need emergency dialysis (14).

Regarding peritoneal dialysis, it was decided that a peritoneal catheter should be placed, despite the fact that the patient was overweight (dry weight 88 kg) and had no residual renal function. This was chosen because there was a deadlock in terms of vascular access, so it was considered that providing "poor dialysis clearance" would be "very good" compared to not providing any form of dialysis at all. However, the first placement of the peritoneal dialysis catheter was unsuccessful, as the catheter entered the right hypochondrium and thus needed repositioning.

The placement of the dialysis catheter by Frampton et al., who were surgeons, was done with the assistance of ultrasound. We, as nephrologists, inserted the dialysis catheter transdermally, without the use of ultrasound, having conducted many such placements during the 1980s for the application of continuous arteriovenous hemofiltration and hemodiafiltration in patients in the intensive care unit. On the day of catheter insertion, our patient had 236,000/mm^3^ platelets and INR = 1.4.

Regarding anticoagulation, in their cases, Frampton et al. heparinized the catheter 24 hours/day (in the form of continuous administration with a pump) with 12,000 IU of classic heparin/lumen, in a dilution with 48 ml 0.9% NaCl (13); Abutaleb performed the same procedure with 4500 tanzaparin subcutaneously once a day (14). Since our patient was difficult and almost never followed our medical instructions, when the catheter was in the femoral artery, a decision was made not to use any anticoagulant (heparin, aspirin, clopidogrel), because it was known that the patient would not be bedridden (as this finally happened), and the chance of catheter movement and bleeding would thus be increased. For the same reason, the catheter lumens were locked using 15% NaCl.

No particular problems were found during the dialysis of the patient. The venous pressure increased only three times (up to 250 mmHg) without any other problems (blood supply 270–300 ml/min). Frampton et al. found that the median dialysis time/session (3.5 hours) and blood supply to the filter (262.5 ml/min) statistically did not differ significantly from the values of those patients who were dialyzed by arteriovenous fistulae or vascular access points other than the femoral artery. Corresponding blood flow levels (200-250 ml/min) were found by Bünger et al. in axillary grafts, which were placed in 20 patients for dialysis (9). There are, of course, potential complications at arterial access points, such as bleeding, hematomas, or pseudoaneurysms, as well as ischemia or embolism of distal arterial branches (15); however, saving the patient's life is paramount. Thus, of the 10 patients Frampton et al. encountered, two had complications. One developed ischemia in the leg three days after catheter insertion, which subsided with the catheter removal, and the other bled because the external pressure on the artery after catheter removal was not sufficiently long (13). We did not observe any complications while the catheter was in the artery; however, 20 days after removal of the catheter, an episode of thrombosis of the common iliac artery was reported, with ischemia in the left leg, which was successfully treated by vascular surgeons. In their patients, Frampton et al. preserved the catheters for an average of five days (from 1–12 days) (13), Abutaleb for two to three months (14), and our patient for 20 days, during which he underwent 11 dialysis sessions. When the catheter was no longer necessary, we preferred surgical removal and restoration of access, while Frampton et al. preferred pressure on the point of insertion of the catheter for only 20 min (13). Based on our experience, we considered acting likewise, using the application of continuous arteriovenous hemofiltration or hemodiafiltration; however, the long stay of the catheter in the artery and the patient's non-compliance to our instructions did not give us the certainty that simple pressure on the artery, even for an extended period of time, would be safe after the catheter removal. In addition, it should be noted that our hospital did not have a vascular surgeon who could treat possible bleeding of the artery after the removal of the catheter. It seems that the use of an artery for the placement of a catheter for dialysis is a solution for patients in whom the placement of a venous dialysis catheter is impossible and peritoneal dialysis is difficult. This approach is easy to apply as well as safe for the patient, and most importantly it provides time to find a solution for dialysis, thus allowing the patient to survive.
